# Gastric-Selective Associations of Caudate Functional Connectivity with Gastrointestinal Rhythms in Parkinson’s Disease: A Resting-State fMRI and Electrogastroenterography Study

**DOI:** 10.3390/brainsci16080823

**Published:** 2026-08-01

**Authors:** Zhining Li, Nana Shen, Can Li, Liangqun Rong, Zhengwei Chen, Chun-Feng Liu

**Affiliations:** 1 Department of Neurology and Clinical Research Center of Neurological Disease, The Second Affiliated Hospital of Soochow University, Suzhou 215004, China; zhining_li@126.com; 2 Department of Neurology, The Second Affiliated Hospital of Xuzhou Medical University, Xuzhou 221006, China; 760020251525@xzhmu.edu.cn (N.S.); jiangsulican@126.com (C.L.); rongliangqun@163.com (L.R.)

**Keywords:** Parkinson’s disease, functional magnetic resonance imaging, electrogastrography, brain–gut axis, functional connectivity

## Abstract

**Highlights:**

**What are the main findings?**

- Functional connectivity of the caudate nuclei is preferentially associated with postprandial gastric, rather than lower intestinal, electrophysiological pacemaking rhythms in Parkinson’s disease.
- Patients with Parkinson’s disease exhibit altered striatal–thalamic network connectivity, with increased thalamo-insular connectivity that correlates with the severity of gastrointestinal non-motor symptoms.

**What are the implications of the main findings?**

- These results provide multimodal objective evidence demonstrating an association between central striatal–thalamic network reorganization and peripheral gastrointestinal autonomic dysfunction.
- Identification of altered functional brain–gastrointestinal relationships provides further insight into potential pathophysiological pathways underlying Parkinsonian non-motor phenotypes and identifies candidate central–visceral network correlates of autonomic dysfunction.

**Abstract:**

**Background/Objectives**: Gastrointestinal dysmotility (GID) is a frequent non-motor manifestation of Parkinson’s disease (PD). However, its peripheral electrophysiological characteristics, as well as the underlying neural mechanisms within the brain–gut axis, remain insufficiently understood. This study aimed to delineate spatiotemporal abnormalities in gastrointestinal pacing activity in PD and to explore their associations with cerebral functional connectivity (FC). **Methods**: Multichannel electrogastroenterography (EGEG) recordings, including both preprandial and postprandial states from gastric (leads 1–4) and intestinal (leads 5–8) regions, were obtained from patients with PD and healthy controls (HCs), alongside resting-state functional MRI (rs-fMRI). The striatal–thalamic circuit was selected as the seed region for FC analysis. Between-group differences in EGEG-derived spatiotemporal metrics were assessed using analysis of covariance (ANCOVA), while FC differences were examined using two-sample *t*-tests. Partial correlation analyses were conducted to evaluate associations among neuroimaging measures, aberrant gastrointestinal electrophysiological indices, and clinical variables. Regional specificity of correlations was further tested by comparing dependent correlation coefficients. In addition, multivariate brain–gut connectivity was assessed using partial canonical correlation analysis (pCCA) with 1000 permutation tests. **Results**: Relative to HCs, PD patients exhibited a significant reduction in the proportion of normal slow waves in both gastric and intestinal regions during preprandial and postprandial states (pFDR < 0.05). FC analysis revealed increased connectivity between the left thalamus and right insula in PD, whereas interhemispheric connectivity of the caudate nuclei and putamina was significantly reduced. Additionally, FC between the left pallidum and left precentral gyrus was attenuated in the PD group. Partial correlation analysis demonstrated a positive association between postprandial normal slow-wave fraction and interhemispheric caudate connectivity (r = 0.63, pFDR = 0.047). Furthermore, left thalamus–right insula connectivity correlated with both total Non-Motor Symptoms Scale (NMSS) scores (r = 0.57, pFDR = 0.042) and gastrointestinal subscale scores (r = 0.63, pFDR = 0.037). At the multivariate level, pCCA revealed a strong association between lentiform nucleus connectivity and preprandial gastric slow-wave rhythms (canonical r = 0.892, *p* = 0.004). **Conclusions**: In PD, caudate nucleus FC shows a preferential association with gastric rather than intestinal electrophysiological activity. Multivariate analyses further demonstrate a significant network-level association between lentiform nucleus connectivity and baseline gastric pacing rhythms, extending regional findings to a broader network interaction. Together, these results provide multimodal correlational evidence reflecting parallel central and peripheral alterations in PD, highlighting synchronized alterations in central networks and peripheral gastrointestinal rhythmicity.

## 1. Introduction

Parkinson’s disease (PD) is a progressive neurodegenerative disorder that imposes a substantial burden on patients, families, and healthcare systems [1]. Although traditionally defined by its cardinal motor manifestations—including resting tremor, bradykinesia, rigidity, and postural instability—the clinical phenotype of PD is now recognized to extend far beyond motor impairment. In particular, a wide range of non-motor symptoms often precede motor onset by years or even decades [2]. Among these non-motor features, gastrointestinal (GI) dysfunction is both highly prevalent and clinically significant. It commonly presents as constipation, delayed gastric emptying, and dysphagia, all of which impair nutrient absorption, reduce the efficacy of dopaminergic therapies, and markedly diminish quality of life [3]. Despite its clinical importance, effective management remains limited. Current treatment strategies, largely based on levodopa replacement and deep brain stimulation, have minimal impact on the underlying gastrointestinal pathology, highlighting a persistent therapeutic gap.

Previous epidemiological data indicate that nearly half of patients present gastrointestinal (GI) complications at the initial diagnosis of Parkinson’s disease (PD) [4], prompting recent expert consensus guidelines to recommend systematic clinical screening [5]. These findings support the “gut-first” hypothesis, which proposes that autonomic GI lesions emerge prior to motor onset [6]. Mechanistically, this bidirectional communication is mediated by the microbiota–gut–brain axis, involving neuroinflammatory cascades, vagal propagation of α-synuclein, and enteric dopaminergic depletion [7,8]. While preclinical interventions targeting the microbiota–gut–brain axis have shown therapeutic potential [9], the exact translational route to human physiology remains unclear. For instance, there is still a lack of direct, quantitative evidence cross-linking central brain network alterations with specific peripheral gastrointestinal electrophysiological rhythms in human PD [10,11,12,13,14]. In addition, most prior assessments rely on subjective symptom scales or indirect markers like transit time, failing to reflect real-time electrophysiological crosstalk between the brain and the gut [15].

Addressing this critical disconnect requires a multimodal approach capable of simultaneously capturing high-resolution central functional connectivity (FC) and precise peripheral myoelectric activity. Rather than relying on indirect symptom-based correlations, such an approach allows for a clearer characterization of the statistical associations between specific neural circuits and gastric pacemaker function. To bridge this gap, the present study adopts a cross-sectional multimodal design integrating resting-state functional magnetic resonance imaging (rs-fMRI) with multichannel surface electrogastroenterography (EGEG). This combined framework allows for non-invasive characterization of intrinsic brain network organization alongside simultaneous assessment of the spatiotemporal dynamics of gastric and intestinal myoelectric rhythms under both fasting and postprandial conditions. In contrast to prior studies that have largely examined central and peripheral measures independently, the current protocol specifically targets the striatal–thalamic network. This circuit is a key hub not only for somatomotor regulation but also for visceral sensory integration and autonomic gastrointestinal control [16,17,18,19], making it particularly relevant for investigating brain–gut interactions in PD. While rs-fMRI provides high-resolution mapping of FC within deep brain structures, EGEG offers an objective and quantitative measure of gastric slow-wave activity and propagation patterns in a fully non-invasive manner [20,21], thereby minimizing reliance on subjective symptom reporting and associated bias. Together, these modalities provide a unique opportunity to interrogate the brain–gut interface in PD from a systems-level perspective, linking central network dysfunction to peripheral electrophysiological disturbances.

The present study was designed with three primary objectives. First, we aimed to characterize and compare the spatiotemporal features of gastrointestinal myoelectric activity between patients with PD and healthy controls (HCs), with particular emphasis on the stability of normal slow-wave rhythms and coordination across proximal and distal intestinal segments. Second, we sought to identify abnormal patterns of FC within the striatal–thalamic network in PD, focusing on regions implicated in visceral sensory integration and autonomic output regulation. Third, we evaluated the associations between central FC measures and peripheral gastrointestinal electrophysiological parameters while adjusting for relevant clinical covariates. We hypothesized that brain–gut interactions in PD exhibit anatomical specificity, whereby disruptions in subcortical connectivity preferentially influence gastric electrical rhythms rather than distal intestinal activity. By delineating these relationships, this study aims to provide further insight into the non-motor pathophysiology in PD and to identify potential imaging–electrophysiological biomarkers that may inform future patient stratification and the development of neuromodulatory strategies aimed at restoring brain–gut homeostasis.

## 2. Materials and Methods

### 2.1. Participants

This study enrolled 25 patients with idiopathic PD and 25 HCs, matched for age, sex, and educational level. PD diagnoses were independently confirmed by senior movement disorder specialists in accordance with the clinical diagnostic criteria of the International Parkinson and Movement Disorder Society (MDS) [19]. All HCs completed the study protocol. Among PD participants, two were excluded: one due to inability to complete data acquisition and one due to excessive motion artifacts that rendered the recordings unusable. Accordingly, the final analytic sample comprised 23 PD patients and 25 HCs. The study protocol was approved by the Institutional Review Board (IRB) and the Clinical Research Ethics Committee of our institution. All procedures were conducted in accordance with the Declaration of Helsinki. Written informed consent was obtained from all participants after a full explanation of the study procedures.

### 2.2. Clinical and Neuropsychological Assessments

All PD patients underwent standardized clinical evaluations prior to neuroimaging and electrophysiological recordings. To ensure temporal consistency, all assessments were completed within one month of the imaging and electrophysiological examinations. Participants were evaluated in the dopaminergic “OFF” state. 

To ensure linguistic accuracy and cross-cultural validity, officially translated and psychometrically validated Chinese versions of all assessment instruments were used. Motor symptom severity was assessed using the Movement Disorder Society-sponsored Unified Parkinson’s Disease Rating Scale Part III (MDS-UPDRS-III) [22]. Non-motor symptoms, with a particular focus on gastrointestinal dysfunction, were evaluated using the Non-Motor Symptoms Scale (NMSS) and its gastrointestinal subscale (NMSS-GI) [23]. Cognitive performance was assessed using Montreal Cognitive Assessment (MoCA) scores extracted from routine clinical records in the hospital electronic medical record (EMR) system [24]. Psychiatric symptoms were evaluated using the Hamilton Anxiety Rating Scale (HAMA) [25] and the Hamilton Depression Rating Scale (HAMD) [26]. Levodopa Equivalent Daily Dose (LEDD) was calculated according to established conversion formulas.

**Figure 1:**
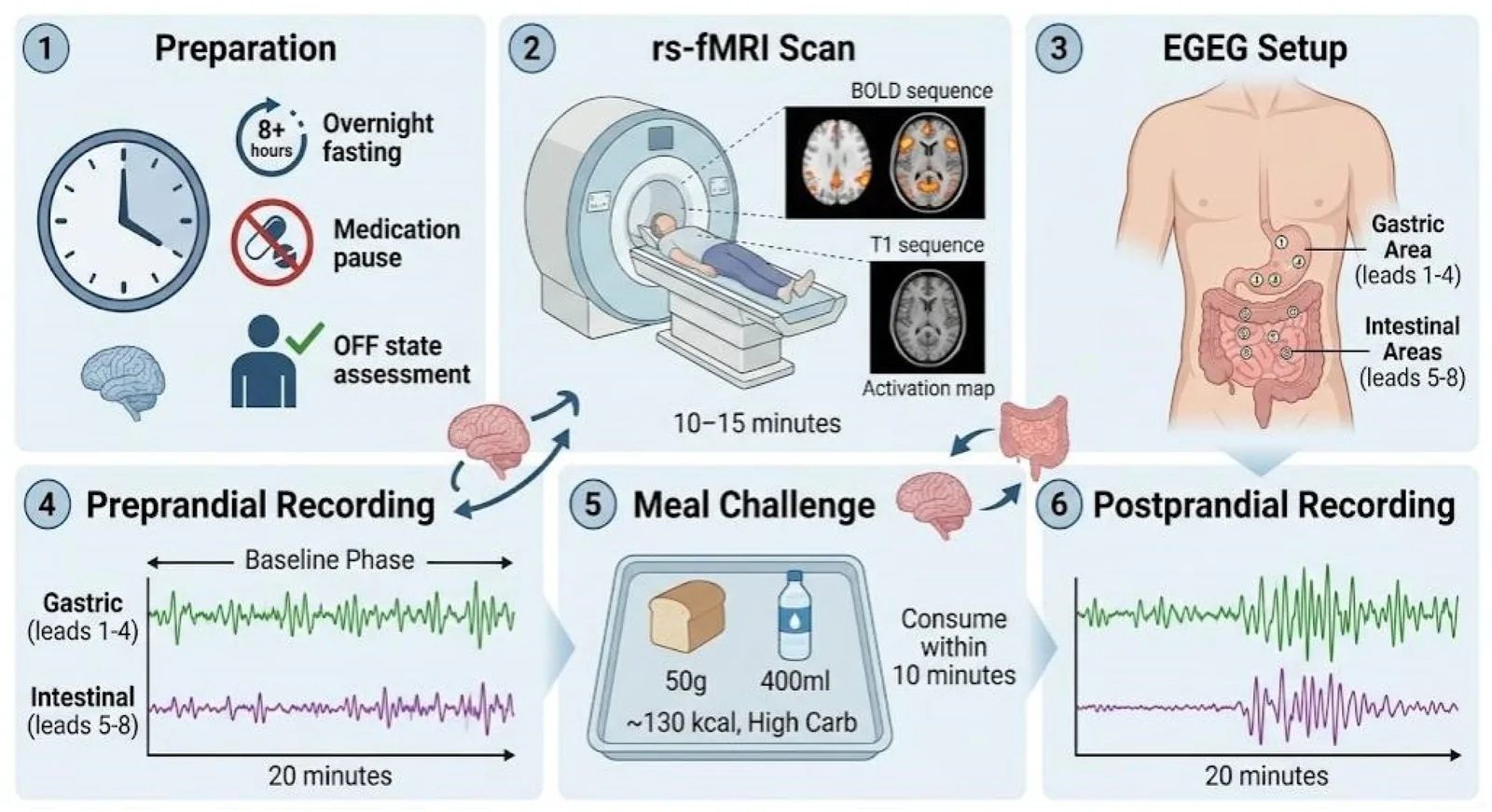
**Schematic illustration of the multimodal experimental design and study** **timeline.**

To ensure reproducibility and clarify the temporal organization of multimodal data acquisition, the overall study workflow and timeline are summarized in Figure 1.

The experimental protocol was conducted in six sequential steps to ensure data integrity and minimize potential confounding effects. (Step 1) Participants underwent an overnight fast of at least 8 h and temporarily discontinued non-essential medications; all assessments were performed in the dopaminergic “OFF” state. (Step 2) Resting-state functional MRI (rs-fMRI), including T1-weighted structural imaging and BOLD sequences, was acquired over 10–15 min, ensuring that neuroimaging measures were not influenced by recent food intake. (Step 3) After skin preparation and degreasing, an 8-channel electrogastroenterography (EGEG) electrode array was applied while participants remained at rest. (Step 4) A 20 min baseline (preprandial) EGEG recording was obtained, capturing gastric (channels 1–4) and intestinal (channels 5–8) myoelectric activity. (Step 5) Participants then consumed a standardized test meal (50 g bread and 400 mL water; ~130 kcal) within 10 min. (Step 6) A subsequent 20 min postprandial EGEG recording was performed to assess gastrointestinal electrophysiological responses to the meal challenge.

### 2.3. Neuroimaging Data Acquisition

Neuroimaging data were acquired using a 3.0-Tesla MRI scanner (Discovery MR750, GE Medical Systems, Milwaukee, WI, USA). During scanning, participants were instructed to remain awake with eyes closed, keep their heads still, and relax without engaging in structured thinking. rs-fMRI data were obtained using an echo-planar imaging (EPI) sequence with the following parameters: repetition time (TR) = 2000 ms, echo time (TE) = 30 ms, flip angle (FA) = 90°, field of view (FOV) = 200 × 200 mm, matrix size = 64 × 64, and slice thickness/gap = 3/0 mm, resulting in 210 consecutive volumes. High-resolution T1-weighted structural images were acquired using a 3D-BRAVO sequence with parameters of TR = 2500 ms, TE = 3.5 ms, FA = 8°, matrix size = 256 × 256, and slice thickness/gap = 1/0 mm, covering 144 slices.

### 2.4. Resting-State fMRI Data Preprocessing

Preprocessing of rs-fMRI data was performed in batch mode using the CONN toolbox (v18b) and SPM12 within MATLAB (R2023a). To ensure methodological rigor and reproducibility, the preprocessing pipeline followed standardized clinical FC mapping guidelines endorsed by the American Society of Functional Neuroradiology [27]. The preprocessing steps included the following: (1) removal of the first 10 volumes to allow for signal stabilization; (2) slice-timing correction to account for temporal differences in slice acquisition; (3) realignment for correction of head motion and identification of excessive movement; (4) outlier detection using the Artifact Detection Tools (ART), with exclusion of frames exceeding a framewise displacement (FD) threshold of 0.9 mm or a global signal threshold of z = 5; (5) correction of image origin offsets resulting from manual positioning; (6) spatial normalization using a two-step procedure, including co-registration of T1 structural images to the mean functional image, followed by tissue segmentation and normalization to a standard tissue probability map (TPM); (7) temporal band-pass filtering (0.01–0.08 Hz) to reduce low-frequency drift and high-frequency physiological noise; (8) linear detrending and removal of abnormal EPI voxels; and (9) regression of nuisance covariates, including head motion parameters, white matter signals, and cerebrospinal fluid signals.

### 2.5. Seed-Based FC Calculation

FC between predefined seed regions and the whole brain was computed using a seed-to-voxel approach. Considering their established roles in visceral sensory processing and autonomic gastrointestinal regulation [16,17,18,19], bilateral thalamus, caudate nucleus, putamen, and pallidum within the striatal–thalamic network were selected as seed regions. These eight regions of interest (ROIs) were extracted from the FSL Harvard–Oxford atlas integrated within the CONN toolbox. For each seed, the mean time series was extracted from the preprocessed functional images. Pearson correlation coefficients were then calculated between each seed region and all brain voxels. To improve normality for statistical analysis, correlation coefficients were transformed into z-scores using Fisher’s r-to-z transformation.

### 2.6. Multichannel EGEG Acquisition and Feature Extraction

**Figure 2:**
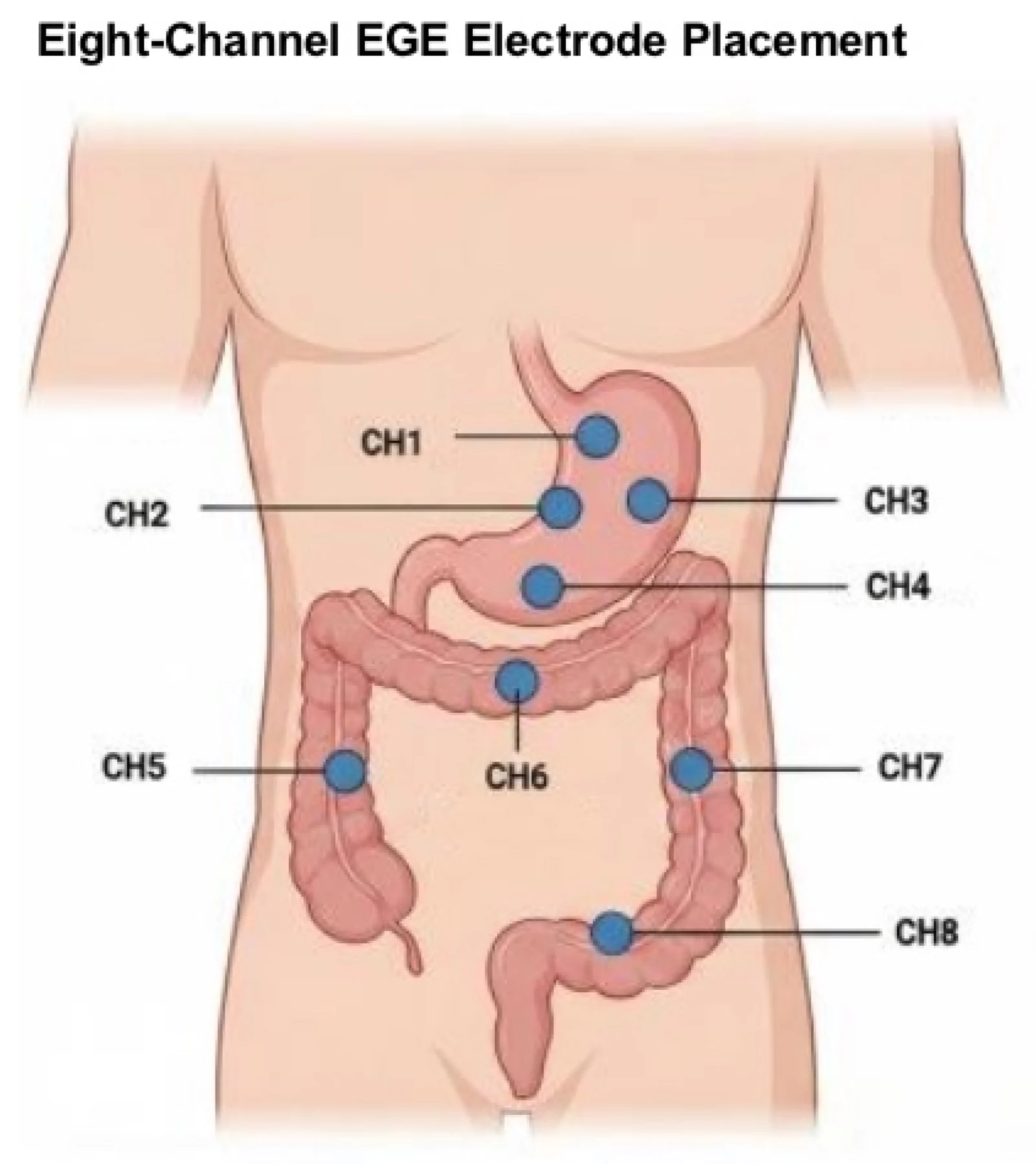
**Schematic illustration of the eight-channel cutaneous electrogastroenterography (EGEG) electrode configuration.** Electrodes were positioned on the abdominal surface according to standardized anatomical landmarks to record myoelectrical activity from distinct gastrointestinal regions. Channels 1–4 were allocated to gastric regions in a proximal-to-distal arrangement: CH1 (gastric body), CH2 (lesser curvature), CH3 (greater curvature), and CH4 (antrum). Channels 5–8 were positioned to capture lower intestinal activity. The reference and ground electrodes were placed at the medial aspect of the right wrist and the medial aspect of the right ankle, respectively.

Gastrointestinal myoelectric activity was continuously recorded using an eight-channel EGEG system (XDJ-S8; Hefei Kaili Co., Hefei, Anhui, China). Participants were instructed to abstain from alcohol and from consuming spicy, greasy, or irritating foods for 72 h prior to recording. On the morning of data acquisition, they followed a clear liquid diet and fasted for at least 8 h, with all medications withheld. Participants were also instructed to avoid vigorous physical activity, smoking, and psychologically stressful stimuli. After careful skin preparation and degreasing with 95% alcohol, eight Ag/AgCl surface electrodes (Hanjie Co., Ltd., Shanghai, China) were placed on the abdominal surface according to standardized topographical guidelines (Figure 2). Skin impedance was confirmed to be below 5 kΩ prior to recording. The reference electrode was positioned at the medial aspect of the right wrist, and the ground electrode was placed on the medial aspect of the right ankle. Channels 1–4 were aligned over projections of the gastric corpus and antrum, while channels 5–8 were positioned more caudally to capture intestinal rhythms. Participants were instructed to remain still and silent throughout the recording. Each session consisted of a 20 min preprandial recording, followed by ingestion of a standardized test meal (50 g bread and 400 mL water consumed within 10 min; approximately 130 kcal, composed of 77% carbohydrates, 12% protein, and 11% fat), and a subsequent 20 min postprandial recording. This low-calorie standardized meal was designed to ensure safety, improve tolerability, and minimize gastrointestinal discomfort, particularly in older PD patients assessed in the dopaminergic OFF state.

Signals were recorded at a sampling rate of 1 Hz and preprocessed using a zero-phase finite impulse response (FIR) band-pass filter (order = 100), with cutoff frequencies of 0.008–0.3 Hz to remove baseline drift and high-frequency physiological noise, including cardiac artifacts. Continuous visual monitoring was performed throughout acquisition to document and flag segments affected by motion, speech, or coughing for subsequent artifact rejection. Artifact removal was conducted using predefined quantitative and visual criteria. (1) Segments exhibiting baseline drift or abrupt voltage shifts exceeding ±300 µV due to gross body movement or respiratory artifacts were excluded from spectral analysis. (2) High-frequency non-physiological spikes, typically associated with muscle contractions or emetic activity, were removed. (3) Only artifact-free continuous segments of at least 2 min were retained, with a minimum of 18 min of usable data required per 20 min recording for fast Fourier transform (FFT) analysis. Gastric and intestinal slow waves were differentiated based on electrode topography and intrinsic frequency characteristics. Channels 1–4 were designated for gastric activity, while channels 5–8 represented intestinal activity (Figure 2). Frequency-based separation further validated regional classification: gastric slow waves were centered at approximately 3 cycles per minute (cpm) with a physiological range of 2.4–3.6 cpm, whereas intestinal slow waves exhibited higher frequencies of 10–15 cpm. For each channel, electrophysiological parameters were extracted independently, including (1) rhythmicity indices (e.g., percentage of normal slow waves and dominant frequency), reflecting the integrity of interstitial cells of Cajal (ICC) pacemaking activity; and (2) spatial conduction measures, including lead-time difference (LTD), which reflects synchronization of electrophysiological propagation across the gastrointestinal smooth muscle network. All derived parameters were cross-validated against established clinical EGEG normative standards to ensure consistency in spectral and conduction-based metrics.

### 2.7. Statistical Analysis

All statistical analyses were performed using SPSS version 26.0 (IBM Corp., Armonk, NY, USA) and Python 3.10, with the Statsmodels (v0.14.0) and SciPy (v1.10.1) libraries. The normality of continuous variables was assessed using both the Shapiro–Wilk and Kolmogorov–Smirnov tests. Categorical variables were summarized as frequencies and percentages (*n*, %), and between-group differences were evaluated using the chi-square test or Fisher’s exact test when expected cell counts were <5. Baseline demographic and clinical characteristics (Table 1) are presented as mean ± standard deviation (SD). In contrast, primary outcome variables used in inferential analyses, including spatiotemporal EGEG metrics (Table 2) and FC z-scores, are reported as mean ± standard error of the mean (SEM) to reflect estimation precision [28]. Homogeneity of variances was assessed using Levene’s test prior to independent sample comparisons. When the assumption of equal variances was satisfied (*p* > 0.05), Student’s *t*-test was applied; otherwise, Welch’s *t*-test was used to control for heteroscedasticity. For non-normally distributed variables, data were expressed as median with interquartile range (IQR) and compared using the Mann–Whitney U test. To jointly evaluate group differences across key baseline variables (age, BMI, education, MoCA, HAMA, HAMD, and mean FD), while controlling for family-wise Type I error inflation, a rank-transformed multivariate analysis of variance (MANOVA) was conducted using the non-parametric bridge approach proposed by Conover and Iman [29]. Specifically, raw data were pooled and converted to ranks prior to multivariate modeling, with group (PD vs. HC) entered as the fixed factor. Within the PD cohort, disease-related clinical variables, including levodopa equivalent daily dose (LEDD), were analyzed descriptively. Statistical significance was defined as *p* < 0.05.

Between-group differences in spatiotemporal EGEG metrics were assessed using ANCOVA, adjusting for age, sex, body mass index (BMI), global cognitive status (derived from electronic medical records), and scores on the HAMA and HAMD. Multiple comparisons were controlled using false discovery rate (FDR) correction, with statistical significance set at pFDR < 0.05. To further ensure robustness and mitigate potential inflation of Type I and Type II errors associated with multiple univariate tests, a multivariate analysis of covariance (MANCOVA) was additionally performed as a system-level validation. Prior to analysis, all continuous EGEG variables were rank-transformed using the Conover–Iman approach [29]. This rank-based MANCOVA model simultaneously assessed group differences in the multivariate EGEG profile while adjusting for the same set of demographic and clinical covariates. Assumptions underlying ANCOVA/MANCOVA were systematically evaluated as follows: (1) homogeneity of regression slopes was tested via Group × covariate interaction terms; (2) homogeneity of variance–covariance matrices was assessed using Box’s M test; and (3) the effects of individual covariates were examined within the omnibus model. Detailed results of these diagnostic tests are provided in Supplementary Table S3.

Within the CONN toolbox, between-group differences in FC were assessed using two-sample *t*-tests, with age, sex, body mass index (BMI), global cognitive status, HAMA, HAMD scores, and mean FD included as nuisance covariates. Statistical significance was defined at the voxel level (*p* < 0.001, uncorrected) and at the cluster level (*p* < 0.05, FDR-corrected, two-tailed). To further control for family-wise Type I error inflation arising from multiple seed-based analyses (eight seeds within the striatal–thalamic network), an additional Bonferroni correction was applied at the seed level. Accordingly, cluster-level results were considered robust only if they survived the adjusted threshold of *p* < 0.05/8 = 0.00625. All reported clusters met this stringent correction criterion. To further validate the global consistency of neuroimaging findings, mean Fisher z-transformed connectivity values extracted from the five significant clusters were entered into a multivariate analysis of covariance (MANCOVA). This model treated the five connectivity measures as a multivariate dependent vector and evaluated the overall group effect (PD vs. HCs) while adjusting for age, sex, BMI, MoCA, HAMA, HAMD, and mean FD. The omnibus MANCOVA demonstrated a strong global group effect (Wilks’ λ = 0.1890, F = 28.3255, *p* < 0.001), with detailed parameter estimates provided in Supplementary Table S4.

For regions showing significant FC differences, mean z-values were extracted for each PD participant and entered into partial correlation analyses with key EGEG parameters (e.g., postprandial normal slow-wave percentage) and clinical scales. All models were adjusted for age, sex, BMI, LEDD, global cognitive status, HAMA, HAMD, and mean FD. Multicollinearity was assessed using the variance inflation factor (VIF) analysis, confirming the absence of significant collinearity among covariates (Table S1). To ensure robustness of regression assumptions, diagnostic tests were performed on model residuals: (1) normality was assessed using the Shapiro–Wilk test; (2) homoscedasticity was evaluated using the Breusch–Pagan test; and (3) influential observations were identified using Cook’s distance, with a conservative exclusion threshold of Di > 1.0. All models satisfied these parametric assumptions. Given the inherent biological dependency among gastrointestinal segments and interconnected brain regions, a global Bonferroni correction across all correlation tests would likely increase Type II error rates. Therefore, FDR correction was applied within each seed-specific testing family. In addition, complementary per-seed Bonferroni corrections were conducted based on the number of tests per seed (adjusted thresholds of *p* < 0.0125 and *p* < 0.0167, respectively;). Notably, key brain–gut and clinical correlations (uncorrected *p* = 0.012) remained significant after these conservative corrections, supporting the robustness of the observed effects. All corrected and uncorrected *p*-values are reported in the corresponding tables in the Section 3. Correlations with pFDR < 0.05 were considered statistically significant. A summary of multiplicity control strategies across the full analytical pipeline is provided in Table S5. An exploratory subgroup analysis stratified by motor symptom lateralization was also performed, with methods and results detailed in the Supplementary Materials.

To address the limitations associated with a relatively modest sample size, effect sizes (partial η^2^) and bootstrapped 95% confidence intervals were reported alongside conventional *p*-values. A detailed post hoc power analysis and sample size justification are provided in Supplementary Text S3. To evaluate the regional specificity of brain–gut electrophysiological associations, dependent correlations derived from the same PD cohort (e.g., caudate–gastric versus caudate–intestinal associations) were compared using Steiger’s Z-test. A two-tailed *p* < 0.05 was considered statistically significant for these dependent correlation comparisons. To control for multiple comparisons across multimodal datasets, a hierarchical correction strategy was implemented. First, FDR correction was applied to regional EGEG parameters, fMRI voxel- and cluster-level results, and correlation analyses. In addition, Bonferroni correction was applied at two analytical levels to ensure stringent family-wise error control: (i) seed-to-voxel analyses (adjusted threshold *p* < 0.05/8 = 0.00625), and (ii) localized correlation analyses using two predefined correction levels (*p* < 0.05/4 = 0.0125 and *p* < 0.05/3 = 0.0167). This layered correction framework effectively minimized inflation of Type I error across the multimodal analytical pipeline.

## 3. Results

### 3.1. Demographic and Clinical Characteristics 

A total of 23 patients with PD and 25 HCs were included in the final analysis. As shown in Table 1, demographic comparisons revealed no significant differences between groups in age, sex distribution, BMI, or educational level. Similarly, no significant between-group differences were observed in clinical measures, including global cognitive status (derived from electronic medical records), as well as HAMA and HAMD scores.

### 3.2. Spatiotemporal Disruptions in Gastrointestinal Myoelectrical Parameters

**Figure 3:**
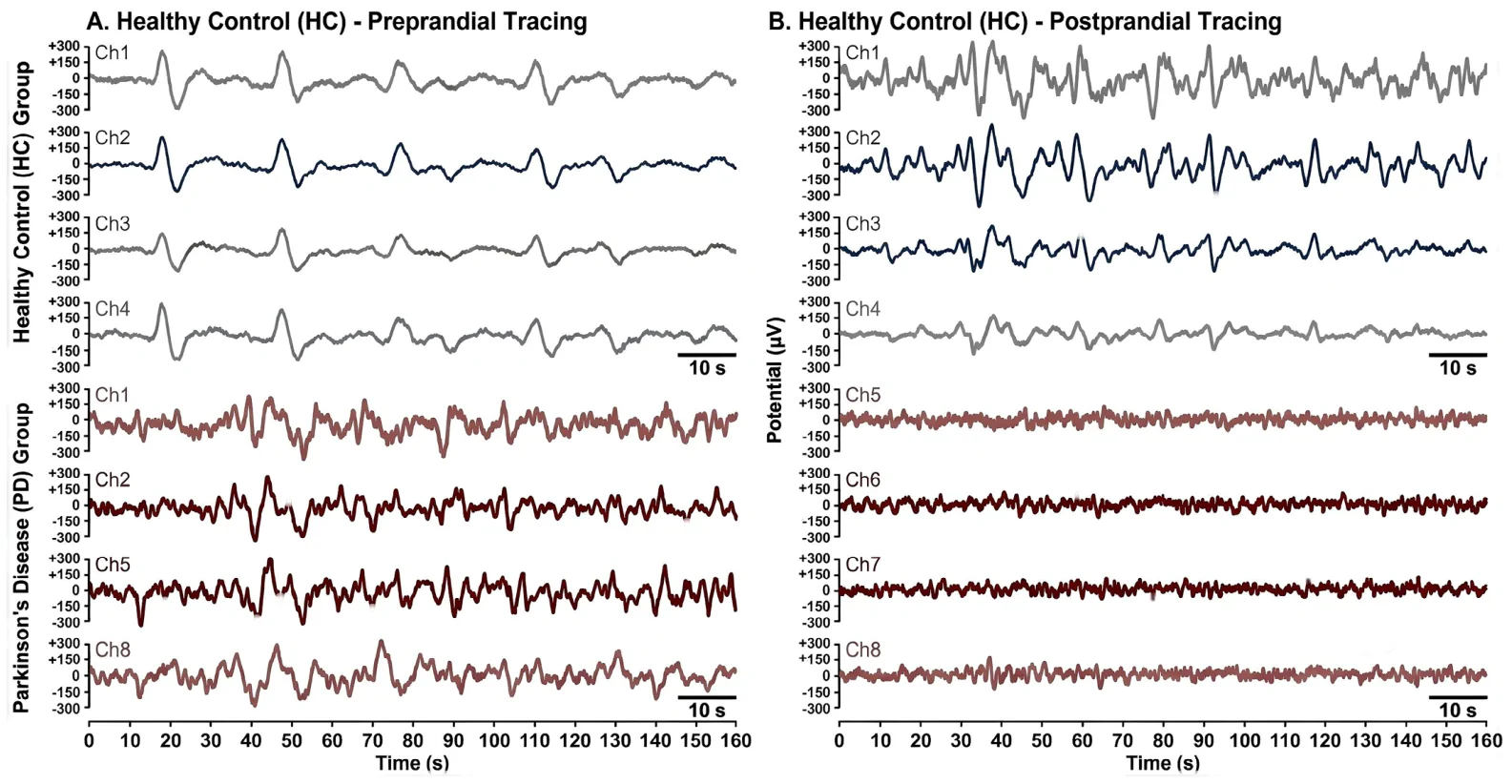
**Representative raw multichannel electrogastroenterography (EGEG) waveforms in healthy controls (HCs) and patients with Parkinson’s disease (PD) under preprandial and postprandial conditions.** (**A**) Preprandial EGEG recording from a representative HC (channels 1–4; green traces). (**B**) Postprandial EGEG recording from the corresponding HC (channels 1–4; green traces), showing a prominent amplitude increase and preserved rhythmicity. (C) Preprandial EGEG recording from a representative PD patient (channels 1, 2, 5, and 8; dark red traces). (D) Postprandial EGEG recording from the corresponding PD patient (channels 1, 2, 5, and 8; dark red traces), demonstrating marked dysrhythmia and blunted postprandial response. The horizontal scale bar indicates 10 s, and the vertical axis indicates electrical potential (μV). Distinct differences in rhythmicity and waveform organization are evident between HC and PD groups, as well as between preprandial and postprandial states.

After rigorous adjustment for age, sex, BMI, global cognitive status, and HAMA/HAMD scores, comparative analyses of key EGEG indices revealed a marked disruption of gastrointestinal pacemaking activity in the PD cohort. This was primarily characterized by a significant reduction in the stability of normal slow-wave activity. Following FDR correction, the proportion of normal slow waves (NSW%) was significantly decreased in both proximal gastric regions (channels 1–4) and distal intestinal regions (channels 5–8) during both preprandial (fasting) and postprandial states in patients with PD compared with HCs (all pFDR < 0.05; Table 2). These quantitative abnormalities were further supported by representative raw EGEG recordings (Figure 3), which demonstrate pronounced dysrhythmia, irregular waveform morphology, and attenuated postprandial amplitude responses in PD. In contrast, HCs exhibited stable, rhythmic, and well-synchronized slow-wave activity across recording channels. 

### 3.3. Modifications in Resting-State FC

**Figure 4:**
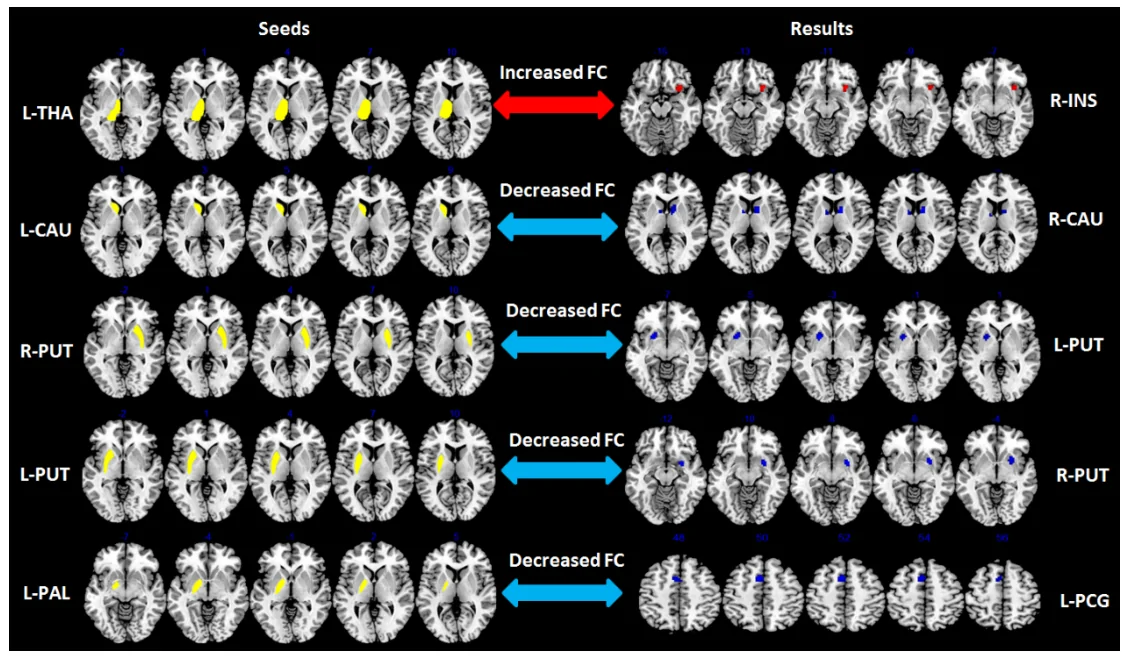
**Spatial distribution of brain regions showing significant functional connectivity (FC) alterations between patients with Parkinson’s disease (PD) and healthy controls (HCs)**. Statistical thresholds were set at the voxel level (*p* < 0.001, uncorrected) and at the cluster level (*p* < 0.05, false discovery rate [FDR]-corrected, two-tailed). Abbreviations: L, left; R, right; THA, thalamus; INS, insula; CAU, caudate; PUT, putamen; PAL, pallidum; PCG, precentral gyrus. Yellow indicates the seed region; red denotes regions showing increased FC in PD compared with HCs; blue indicates regions showing decreased FC in PD compared with HCs.

**Figure 5:**
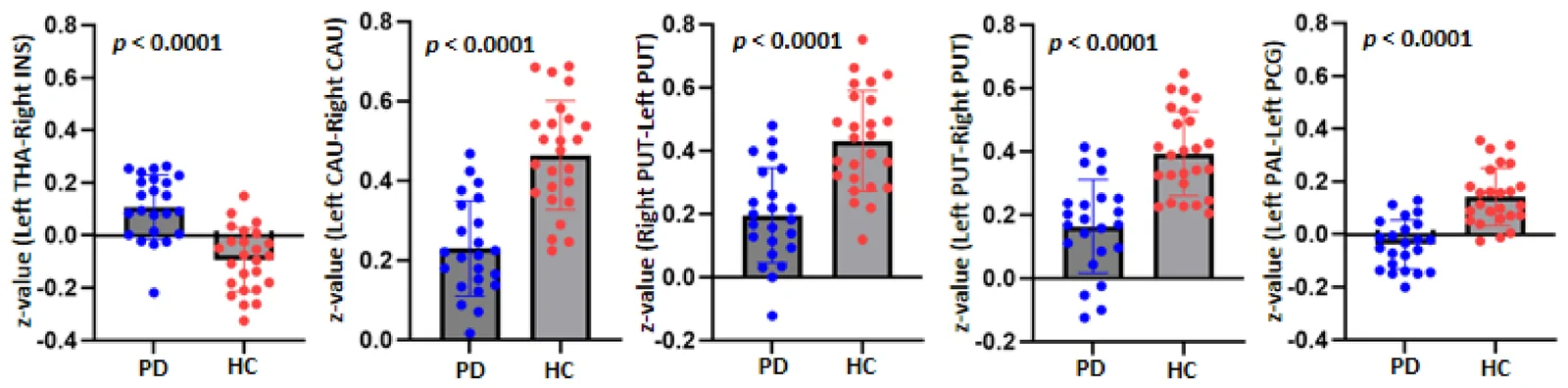
**Functional connectivity values (z-transformed) in patients with Parkinson’s disease (PD) and healthy controls (HCs).** Bar charts overlaid with individual data points depict mean and individual z-values extracted from five pathways showing significant between-group differences. Compared with healthy controls (red dots), patients with PD (blue dots) exhibited significantly increased functional connectivity in the left thalamus-right insula pathway (Left THA−Right INS). In contrast, significantly reduced connectivity was observed in the interhemispheric caudate (Left CAU−Right CAU), reciprocal interhemispheric putamen pathways (Right PUT−Left PUT and Left PUT−Right PUT), and the left pallidum-left precentral gyrus (Left PAL−Left PCG) circuits. Gray bars and error bars indicate group means and standard deviations, respectively. All between-group differences are statistically significant (*p* < 0.0001Abbreviations: THA, thalamus; INS, insula; CAU, caudate; PUT, putamen; PAL, pallidum; PCG, precentral gyrus.

Compared with HCs, patients with PD exhibited significantly increased FC between the left thalamus and the right insular cortex (voxel-level *p* < 0.001; cluster-level *p* < 0.05; Table 3; Figure 4 and Figure 5). In contrast, reduced intra-striatal connectivity was observed bilaterally, including decreased FC between the left and right caudate nuclei and between the left and right putamen (voxel-level *p* < 0.001; cluster-level *p* < 0.05; Table 3; Figure 4 and Figure 5). In addition, FC between the left pallidum and the left precentral gyrus was significantly decreased in the PD group (voxel-level *p* < 0.001; cluster-level *p* < 0.05; Table 3; Figure 4 and Figure 5). Exploratory analyses examining the potential influence of motor symptom lateralization on FC alterations are presented in Supplementary Text S1 and Table S2.

### 3.4. Outcomes of Partial Correlation Analysis 

Prior to correlation analyses, collinearity diagnostics confirmed the absence of significant multicollinearity among covariates, with all VIF values well below the conservative threshold of 5 (range: 1.31–2.41; Supplementary Table S1). 

**Figure 6:**
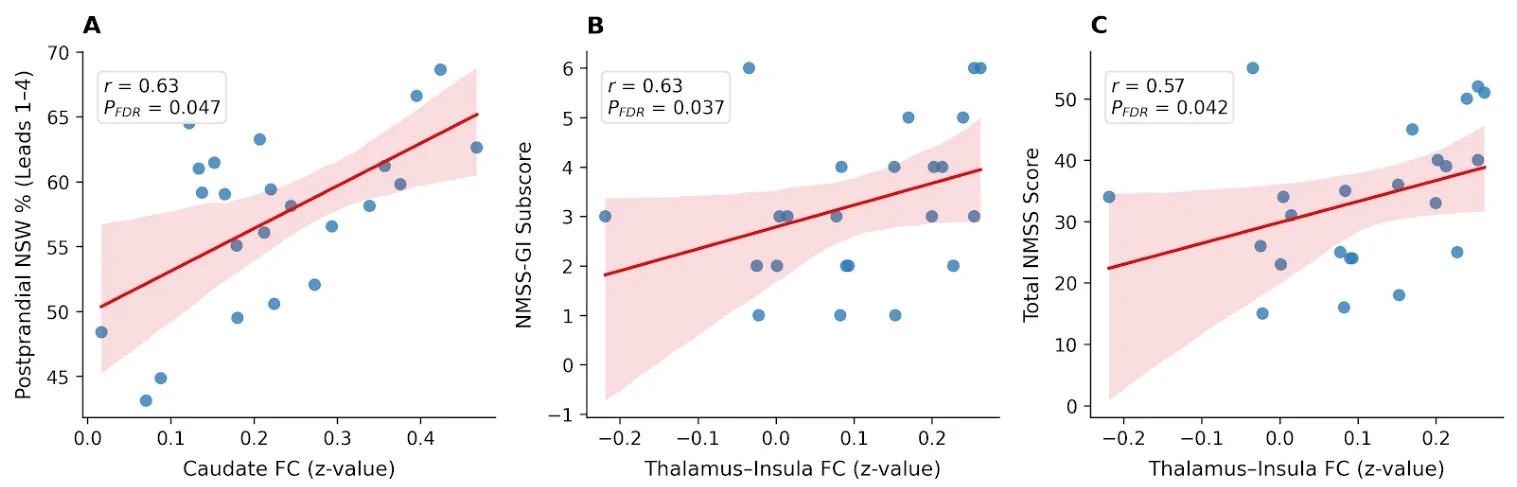
**Significant associations between functional connectivity (FC), gastrointestinal electrophysiological activity, and clinical severity measures in patients with Parkinson’s disease (PD).** (**A**) FC of the left caudate nucleus is positively correlated with the percentage of normal postprandial gastric slow waves (NSW%) (r = 0.630, pFDR = 0.047). (**B**) Functional connectivity of the left thalamus shows a positive association with the gastrointestinal subscore of the Non-Motor Symptoms Scale (NMSS−GI) (r = 0.627, pFDR = 0.037). (**C**) Left thalamic functional connectivity is significantly correlated with total NMSS scores (r = 0.566, pFDR = 0.042). Shaded regions indicate 95% confidence intervals of the regression fits. Abbreviations: BMI, body mass index; FC, functional connectivity; FDR, false discovery rate; HAMA, Hamilton Anxiety Rating Scale; HAMD, Hamilton Depression Rating Scale; L−CAU, left caudate; L−THA, left thalamus; LEDD, levodopa equivalent daily dose; Mean FD, mean framewise displacement; MoCA, Montreal Cognitive Assessment; NMSS, Non-Motor Symptoms Scale; NMSS−GI, Non-Motor Symptoms Scale gastrointestinal subscale score; NSW, normal slow wave. (Note: All models were adjusted for the listed clinical covariates in the underlying partial correlation analyses).

Partial correlation analyses revealed a significant positive association between FC (z-values) of the bilateral caudate nuclei and the percentage of normal postprandial slow waves in the proximal stomach (leads 1–4) (r = 0.63, 95% CI [0.015, 0.930], pFDR = 0.047; Table 4; Figure 6A). In contrast, no significant association was observed in the preprandial state, which showed a non-significant negative trend (r = −0.37, pFDR = 0.346; Table 4). To further examine spatial specificity, this relationship was contrasted with the correlation between inter-caudate FC and postprandial normal slow-wave percentage in the distal intestinal region (leads 5–8), which was not significant (r = 0.18, 95% CI [−0.731, 0.731], pFDR = 0.731; Table 4). Additionally, FC between the left thalamus and right insula was positively associated with both total Non-Motor Symptoms Scale (NMSS) scores (r = 0.57, 95% CI [0.011, 0.924], pFDR = 0.042; Table 5; Figure 6C) and NMSS gastrointestinal subscale scores (r = 0.63, 95% CI [0.052, 0.950], pFDR = 0.037; Table 5; Figure 6B). However, no significant associations were observed between FC measures and MDS-UPDRS-III scores, indicating motor symptom severity (all pFDR > 0.05). Similarly, normal slow-wave percentages in the distal gastrointestinal region (leads 5–8) showed no significant correlations with either clinical scales or FC metrics (all pFDR > 0.05; Table 5). 

### 3.5. Multidimensional Brain–Gut Network Associations via Partial Canonical Correlation Analysis (pCCA)

While the bivariate partial correlation analyses identified specific pairwise associations between discrete brain regions and individual electrophysiological or clinical measures, they were not sufficient to capture higher-order, multivariate interactions between subcortical networks and the global gastric electrophysiological profile. To address this limitation, we performed pCCA within the PD cohort (N = 23), with adjustment for eight covariates (age, sex, BMI, LEDD, MoCA, HAMA, HAMD, and mean FD).

**Figure 7:**
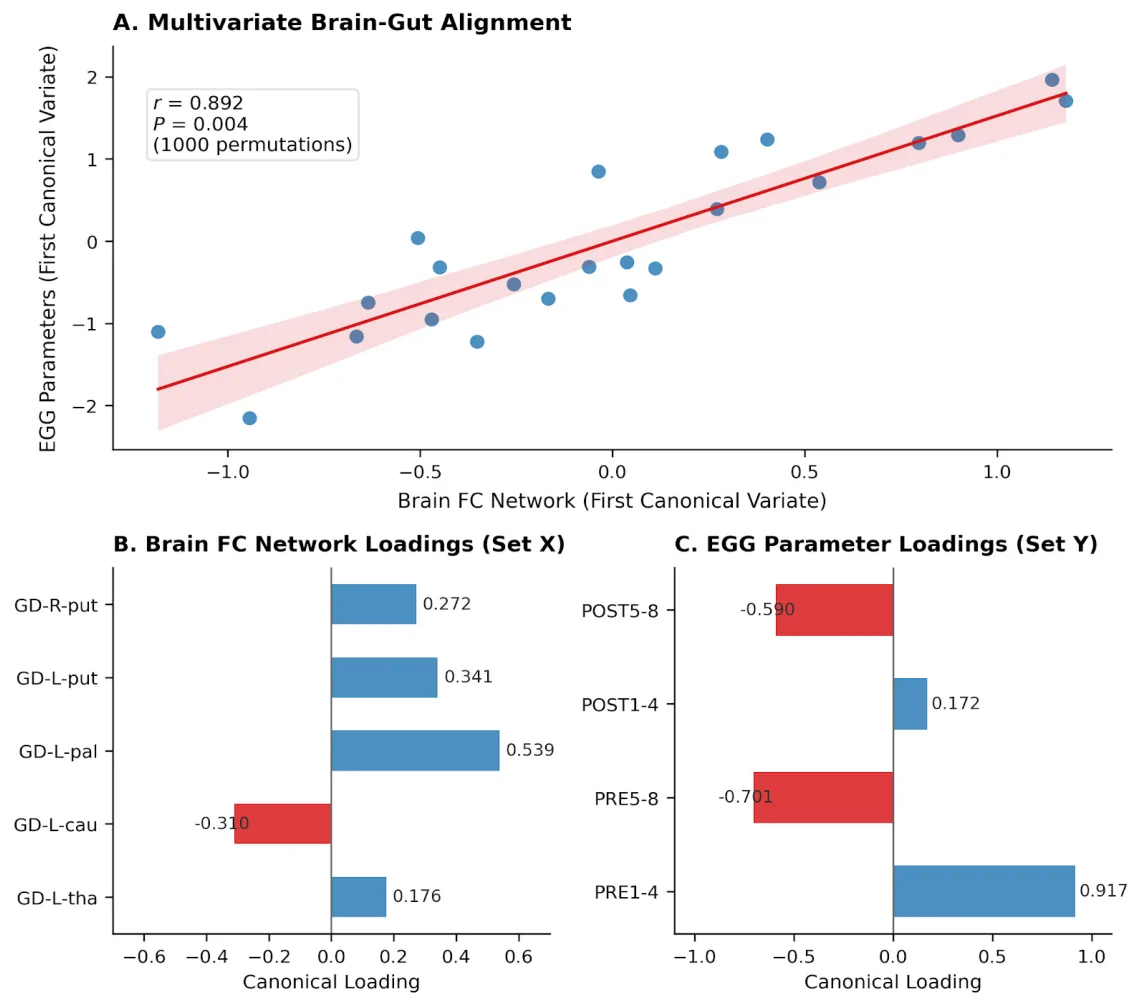
**Partial canonical correlation analysis (pCCA) between brain functional connectivity and electrogastroenterography (EGEG) spatiotemporal parameters.** (**A**) Scatter plot illustrating multivariate network-level association for the first canonical variate pair after adjusting for clinical covariates (age, sex, BMI, LEDD, MoCA, HAMA, HAMD, and mean FD). Each blue dot represents an individual patient (N = 23), and the red line indicates the linear regression fit with the 95% confidence interval. (**B**) Canonical loadings for the five basal ganglia and thalamic seed regions (Set X). (**C**) Canonical loadings for the four EGEG spatiotemporal parameters (Set Y). Blue and red bars represent positive and negative loadings, respectively. The first canonical correlation was statistically significant based on a 1000-time non-parametric permutation test (*p* < 0.01). Abbreviations: BMI, body mass index; EGG, electrogastrography; HAMA, Hamilton Anxiety Rating Scale; HAMD, Hamilton Depression Rating Scale; LEDD, levodopa equivalent daily dose; Mean FD, mean framewise displacement; MoCA, Montreal Cognitive Assessment; pCCA, partial canonical correlation analysis.

A 1000-iteration non-parametric permutation test identified a single statistically significant canonical variate pair (Table 6; Figure 7A), with a strong canonical correlation (r = 0.892, *p* = 0.004). No additional higher-order canonical roots reached statistical significance (all *p* > 0.05), indicating a dominant single mode of brain–gut association. Canonical loading analyses further revealed that this multivariate association was primarily driven by FC within the left pallidum (loading = 0.539) and left putamen (loading = 0.341) in the neural dataset (Set X), which closely aligned with the preprandial normal slow-wave percentage of gastric channels 1−4 (PRE1−4, loading = 0.917) in the electrophysiological dataset (Set Y) (Figure 7B,C).

**Table 4:** Associations between core EGEG metrics and functional connectivity in PD.

FC (z-Values)	Core EGEG Metric	r	*p*_unc_-Value	*p*_FDR_-Value	95% CI (1k-Boot)
Left CAU–Right CAU	Postprandial NSW % (channels 1–4)	0.63	0.012	**0.047**	[0.015, 0.930]
Left CAU–Right CAU	Preprandial NSW % (channels 1–4)	−0.37	0.173	0.346	[−0.915, 0.438]
Left CAU–Right CAU	Postprandial NSW % (channels 5–8)	0.18	0.532	0.731	[−0.476, 0.681]
Left CAU–Right CAU	Preprandial NSW % (channels 5–8)	0.06	0.820	0.820	[−0.650, 1.000]
Left PUT–Right PUT	Postprandial NSW % (channels 1–4)	−0.41	0.126	0.169	[−0.840, 0.545]
Left PUT–Right PUT	Preprandial NSW % (channels 5–8)	0.55	0.034	0.130	[−0.315, 0.950]
Left PUT–Right PUT	Postprandial NSW % (channels 5–8)	−0.49	0.065	0.130	[−0.873, 0.369]
Left PUT–Right PUT	Preprandial NSW % (channels 1–4)	0.13	0.637	0.637	[−0.737, 0.747]
Right PUT–Left PUT	Postprandial NSW % (channels 1–4)	0.07	0.813	0.813	[−0.288, 0.402]
Right PUT–Left PUT	Preprandial NSW % (channels 5–8)	−0.46	0.074	0.147	[−0.171, 0.675]
Right PUT–Left PUT	Postprandial NSW % (channels 5–8)	−0.43	0.075	0.147	[−0.882, 0.220]
Right PUT–Left PUT	Preprandial NSW % (channels 1–4)	0.51	0.110	0.147	[−0.844, 0.205]
Left PAL–Left PCG	Preprandial NSW % (channels 1–4)	0.46	0.082	0.297	[−0.309, 0.914]
Left PAL–Left PCG	Postprandial NSW % (channels 1–4)	0.39	0.148	0.297	[−0.327, 0.901]
Left PAL–Left PCG	Preprandial NSW % (channels 5–8)	−0.24	0.387	0.516	[−0.892, 0.526]
Left PAL–Left PCG	Postprandial NSW % (channels 5–8)	−0.14	0.629	0.629	[−0.827, 0.542]
Left THA–Right INS	Postprandial NSW % (channels 1–4)	0.32	0.248	0.824	[−0.499, 0.866]
Left THA–Right INS	Postprandial NSW % (channels 5–8)	−0.12	0.678	0.824	[−0.805, 0.687]
Left THA–Right INS	Preprandial NSW % (channels 1–4)	0.16	0.824	0.824	[−0.753, 0.786]
Left THA–Right INS	Preprandial NSW % (channels 5–8)	−0.06	0.566	0.824	[−0.537, 0.792]

Note: The pFDR values denote significance levels adjusted for multiple comparisons via the false discovery rate (FDR) method within each independent seed (m = 4 tests per seed). The punc-values denote the uncorrected *p*-value. Additionally, a formal statistical comparison using Steiger’s Z-test between the postprandial Caudate–Gastric correlation (r = 0.63) and the Caudate–Intestinal correlation (r = 0.18) yielded a significant difference (*p* = 0.006), confirming the spatial specificity of this observed functional association. Statistically significant pFDR-values (<0.05) are highlighted in bold. Abbreviations: EGEG, electrogastroenterography; FC, functional connectivity; PD, Parkinson’s disease; NSW, normal slow wave; CAU, caudate; PUT, putamen; PAL, pallidum; THA, thalamus; INS, insula; PCG, precentral gyrus.

**Table 5:** Relationships between functional connectivity and clinical severity scores in PD.

FC (z-Values)	Clinical Scales	r	*p*_unc_-Value	*p*_FDR_-Value	95% CI (1k-Boot)
Left THA–Right INS	NMSS-GI subscore	0.63	0.012	**0.037**	**[0.052, 0.950]**
Left THA–Right INS	NMSS total score	0.57	0.028	**0.042**	**[0.011, 0.924]**
Left THA–Right INS	MDS-UPDRS-III score	0.37	0.179	0.179	[−0.431, 0.883]
Left PAL–Left PCG	NMSS-GI subscore	0.44	0.104	0.281	[−0.285, 0.922]
Left PAL–Left PCG	NMSS total score	0.36	0.187	0.281	[−0.507, 0.871]
Left PAL–Left PCG	MDS-UPDRS-III score	−0.11	0.691	0.691	[−0.756, 0.754]
Left CAU–Right CAU	MDS-UPDRS-III score	0.15	0.603	0.753	[−0.598, 0.772]
Left CAU–Right CAU	NMSS-GI subscore	0.09	0.753	0.753	[−0.589, 0.735]
Left CAU–Right CAU	NMSS total score	0.31	0.265	0.753	[−0.453, 0.882]
Right PUT–Left PUT	MDS-UPDRS-III score	0.21	0.448	0.587	[−0.377, 0.832]
Right PUT–Left PUT	NMSS-GI subscore	0.15	0.587	0.587	[−0.517, 0.799]
Right PUT–Left PUT	NMSS total score	0.51	0.054	0.161	[−0.479, 0.941]
Left PUT–Right PUT	MDS-UPDRS-III score	0.15	0.583	0.817	[−0.469, 0.842]
Left PUT–Right PUT	NMSS-GI subscore	0.07	0.817	0.817	[−0.535, 0.742]
Left PUT–Right PUT	NMSS total score	0.49	0.065	0.195	[−0.595, 0.911]

Note: pFDR-value represents the *p*-value corrected for multiple comparisons using the false discovery rate (FDR) method within each independent seed (m = 3 tests per seed). Punc-value, *p*-value without correction. Partial correlation analyses rigorously controlled for age, sex, body mass index (BMI), levodopa equivalent daily dose (LEDD), Hamilton Anxiety Rating Scale (HAMA), Hamilton Depression Rating Scale (HAMD), global cognitive status (MoCA) and mean framewise displacement (Mean FD). The 95% Confidence Intervals (CI) were generated using 1000 bootstrap resamples. Statistically significant pFDR-values (<0.05) are highlighted in bold. Abbreviations: FC, Functional connectivity; PD, Parkinson’s disease; THA, Thalamus; INS, Insula; PAL, Pallidum; PCG, Precentral gyrus; CAU, Caudate; PUT, Putamen; NMSS, Non-Motor Symptoms Scale.

**Table 6:** Multidimensional network linkage between brain functional connectivity and EGG spatiotemporal parameters via pCCA.

Canonical Variate Pairs	Canonical Correlation (r)	*p* Value
First Canonical Root (Root 1)	0.892	**0.004**
Second Canonical Root (Root 2)	0.809	0.158
Third Canonical Root (Root 3)	0.686	0.432
Fourth Canonical Root (Root 4)	0.133	0.941

Note. Partial Canonical Correlation Analysis (pCCA) was utilized within the PD cohort (N = 23). Confounding clinical factors, including age, sex, body mass index (BMI), levodopa equivalent daily dose (LEDD), MoCA, HAMA, HAMD scores, and mean framewise displacement (Mean FD), were rigorously regressed out to obtain residual matrices prior to the CCA. Significance was evaluated using a 1000-time non-parametric permutation test. *p* < 0.01 (two-tailed). Statistically significant pFDR-values (<0.05) are highlighted in bold.

## 4. Discussion

This study employed a multimodal framework integrating rs-fMRI with multichannel EGEG to simultaneously characterize central FC and peripheral gastrointestinal myoelectric activity. We identified distinct alterations in striatal–thalamic connectivity occurring in parallel with widespread disruptions in gastrointestinal pacemaking rhythms. Consistent with our initial hypothesis, these central network abnormalities were preferentially associated with gastric slow-wave activity rather than intestinal rhythms. This anatomical specificity supports a selective relationship between subcortical circuit variance and localized peripheral electrophysiological impairment, highlighting a potential topographic vulnerability of gastric autonomic regulation in the early-to-mid stages of PD. In addition, these central–visceral associations correlated significantly with overall non-motor symptom burden while remaining independent of motor symptom severity. At the multivariate level, partial canonical correlation analysis (pCCA) demonstrated that this subcortical–visceral relationship extends beyond isolated pairwise features, suggesting a significant network-level covariance between baseline gastric rhythmicity and the intrinsic functional integrity of the lentiform network.

The observed reduction in normal gastrointestinal slow waves provides objective electrophysiological evidence of autonomic dysfunction in PD, extending subjective symptom-based findings [4] and is consistent with models of early neurodegeneration within the enteric nervous system [30]. Disruption of slow-wave rhythmicity likely reflects intrinsic alterations in the interstitial cells of Cajal (ICC) and ENS circuitry [31]. Taken together, peripheral electrophysiological abnormalities may alter the electrophysiological framework associated with delayed gastric emptying commonly observed in clinical settings.

Centrally, the striatal–thalamic network demonstrated pronounced functional reorganization, characterized by thalamo-insular hyperconnectivity alongside intra-striatal hypoconnectivity. Given the insula’s established role in interoceptive processing and autonomic integration [32,33], the increased thalamo-insular connectivity may reflect a maladaptive amplification of aberrant visceral afferent signaling originating from gastrointestinal dysfunction. This interpretation is consistent with emerging evidence suggesting that intestinal barrier disruption can induce central neuroinflammatory responses through ascending vagal pathways [34].

In parallel, reduced connectivity within the bilateral caudate nuclei and putamen is consistent with the canonical dopaminergic degeneration observed in PD. Importantly, our findings extend these well-established motor-related alterations by directly linking striatal network dysfunction to non-motor gastrointestinal phenotypes. Although structural basal ganglia atrophy has been extensively documented [12,35], our results suggest that functional decoupling within intrinsic striatal networks may occur independently of overt morphological changes, thereby contributing to impaired autonomic and visceral regulation [36]. Furthermore, reduced connectivity between the pallidum and the precentral gyrus highlights disruption of cortico–basal ganglia circuits that coordinate both motor output and central autonomic control of gastrointestinal function. Collectively, these connectivity patterns suggest altered functional relationships within gut–brain pathways [37], providing a central neural signature that parallels peripheral electrophysiological dysrhythmia. Although the present macroscale findings cannot resolve microscale mechanisms, whether these subcortical network disruptions interact with upstream post-transcriptional pathways, such as alternative splicing of dopamine receptors or α-synuclein variants, remains an intriguing target for future multi-scale biological validation [38].

Our findings reveal a hierarchical pattern of subcortical–gastric functional relationships in PD across both univariate and multivariate levels. Local partial correlation analyses demonstrated that inter-caudate FC was selectively associated with postprandial, but not preprandial, normal gastric slow-wave percentage (NSW%) (Table 4; Figure 6A). This association showed clear spatial specificity, being confined to gastric rather than distal intestinal measures (*p* = 0.006). Given the caudate nucleus’s involvement in interoceptive integration, cognitive processing, and visceral feedback regulation, this postprandial association may reflect a state-dependent coordination between striatal circuits and digestive phase transitions, potentially linked to dopaminergic modulation of vagal autonomic output during gastric accommodation [8].

In contrast, multivariate pCCA revealed a broader system-level network association predominantly expressed at baseline (preprandial) conditions, centered on the lentiform nucleus (left pallidum and left putamen) and synchronized with fasting gastric NSW% (Table 6; Figure 7). The dissociation between postprandial caudate-specific associations and preprandial lentiform-dominant relationships suggests a complementary, state-dependent organizational pattern of brain–gut dysfunction. Specifically, the putamen and pallidum—key input and output nodes of basal ganglia circuits—appear to exhibit resting-state functional decoupling associated with baseline gastric dysrhythmia, whereas caudate connectivity is selectively engaged under postprandial metabolic challenge, revealing a dynamic modulation of striatal–gut interactions across physiological states.

Correlations between thalamo-insular hyperconnectivity and both global NMSS scores and NMSS gastrointestinal subscale scores further underscore the clinical relevance of these subcortical circuits (Table 5; Figure 6B,C). These associations provide a direct link between objective measures of altered brain connectivity and patient-reported gastrointestinal symptom burden. Importantly, no significant relationships were observed between these connectivity measures and MDS-UPDRS-III motor scores, suggesting that the neural substrates underlying autonomic dysfunction are at least partially distinct from those primarily governing nigrostriatal motor impairment [39]. After adjusting for anxiety and depressive symptoms, these associations remained significant, indicating that the observed brain–gut relationships are unlikely to be driven by mood-related confounding. Collectively, these findings provide converging multimodal evidence supporting a direct association between central network alterations and peripheral gastrointestinal dysfunction in PD. These results are consistent with a growing body of literature across neuropsychiatric and neurodegenerative disorders demonstrating coordinated alterations in brain network organization and peripheral physiological states along brain–body signaling axes. For example, a recent large-scale multimodal fusion study in depression reported that widespread alterations in large-scale brain networks were associated with systemic metabolic dysregulation, including abnormal BMI and disrupted pancreatic endocrine function [40].

Additionally, exploratory subgroup analyses examined the potential influence of motor symptom lateralization on the observed outcomes (Supplementary Text S1; Table S2). Although preliminary differences were observed between subgroups in patterns potentially related to unilateral motor and visceral processing pathways, inter-caudate FC—the primary neural correlate associated with gastric electrophysiological activity—did not differ substantially according to disease onset side. However, given the limited sample size of the subgroups (*n* = 9 vs. *n* = 14), these analyses were underpowered, and the findings should be interpreted as exploratory. Further validation in larger cohorts is therefore required. Finally, all participants were assessed in the dopaminergic OFF state, which enhances the interpretability of intrinsic disease-related network alterations by minimizing acute medication effects. However, this also limits direct translational inference to clinical treatment settings. From a mechanistic perspective, levodopa restores striatal dopaminergic signaling and is known to rapidly modulate cortico-striatal connectivity. In parallel, dopamine exerts significant peripheral effects on gastrointestinal function, including D2 receptor-mediated inhibition of gastric motility and modulation of slow-wave activity. Consequently, transitions between OFF and ON medication states likely induce dynamic reconfiguration of the brain–gut axis. Longitudinal studies incorporating both medication states will therefore be necessary to determine whether the striatal–gastric relationship may serve as a sensitive biomarker for treatment response.

Furthermore, several potential confounding factors should be considered. Although assessment of patients in the dopaminergic “OFF” state minimizes the influence of acute pharmacological effects, long-term dopaminergic therapy may still induce persistent neuroplastic adaptations that cannot be fully disentangled from resting-state subcortical connectivity patterns. In addition, clinical heterogeneity—including motor subtypes (e.g., tremor-dominant versus akinetic-rigid) and variations in disease duration—may contribute to variability in both central FC and peripheral gastrointestinal electrophysiological activity. Progressive disease burden, often accompanied by increasing α-synuclein deposition within both the ENS and central autonomic regulatory regions, may concurrently impair gastric slow-wave stability and alter striatal–thalamic functional organization through disrupted visceral afferent signaling. Moreover, inter-individual differences in baseline autonomic function and constipation severity, reflecting variable involvement of parasympathetic and sympathetic regulation, may further contribute to heterogeneity in gastric pacemaking dynamics. Although key demographic and clinical variables were included as covariates in multivariate models, the complex interrelationships among these factors underscore the multidimensional nature of the Parkinsonian brain–gut axis. These considerations highlight the need for larger, phenotypically stratified cohorts in future studies to better disentangle subtype-specific and disease-stage-dependent effects.

Several limitations of this study should be acknowledged. First, due to its cross-sectional and observational design, the present study is limited to identifying multilevel associative relationships and concurrent functional associations, without permitting inference of causal directionality or temporal sequencing between striatal–thalamic network alterations and peripheral gastrointestinal dysrhythmia. Although our findings delineate a tightly coupled brain–gut association, they do not clarify whether central network reorganization precedes enteric dysfunction, follows it, or reflects parallel pathological processes. Longitudinal and interventional studies, including non-invasive neuromodulation approaches, are therefore required to establish the causal mechanisms underlying these associations. Second, while surface EGEG provides a non-invasive means of assessing gastrointestinal myoelectric activity, it is inherently limited by relatively low spatial resolution and susceptibility to artifacts arising from abdominal wall motion. In addition, as a surface-based technique, EGEG is influenced by volume conduction effects and potential signal overlap between adjacent gastrointestinal structures. Although the 8-channel electrode array was positioned according to standardized anatomical landmarks, and gastric (≈3 cpm) and intestinal (10–15 cpm) rhythms were differentiated using frequency-specific bandpass filtering, signal dispersion through abdominal tissues may still introduce spatial ambiguity. Without concurrent invasive validation methods, such as intraluminal electrodes or manometry, precise localization of pacemaking sources remains limited. Although BMI was included as a covariate to partially account for adiposity-related variability, these methodological constraints suggest that intestinal findings should be interpreted cautiously and warrant further validation using complementary physiological approaches. 

Third, the relatively small sample size of the PD cohort (N = 23) limits the generalizability of the findings. Nevertheless, the robustness of the primary brain–gut association results is supported by moderate-to-large effect sizes (r = 0.63; partial η^2^ > 0.14 for key EGEG group differences), and post hoc power analysis indicated a statistical power exceeding 91% (Supplementary Text S3). Despite this, independent replication in larger, prospectively designed cohorts is essential to confirm the stability of these subcortical–autonomic signatures. Fourth, the standardized meal challenge (50 g bread and 400 mL water; ~130 kcal) represents a relatively mild physiological stimulus compared with conventional high-calorie or high-fat test meals used in gastroenterological assessments. However, this low-calorie protocol was deliberately selected to maximize tolerability and safety in elderly and clinically vulnerable PD patients examined in the dopaminergic “OFF” state. Finally, although multiple clinical covariates were adjusted for, residual confounding from unmeasured factors—such as concomitant non-neurological medications and clinical subtype heterogeneity—cannot be fully excluded.

Future studies should incorporate multi-omics strategies, including gut microbiome metagenomic profiling, to further elucidate the complex interplay within the brain–gut axis. Integrating central neuroimaging, peripheral electrophysiology, and microbial composition data will enable a more comprehensive mechanistic understanding of gastrointestinal dysfunction in PD, ultimately advancing toward a unified systems-level model of brain–gut pathophysiology.

## 5. Conclusions

This study provides multimodal correlational evidence of an association between striatal–thalamic network alterations, objective gastric dysrhythmias, and subjective gastrointestinal symptom burden in PD, independent of motor severity. Notably, inter-caudate FC, rather than putamen or pallidum connectivity, showed a preferential association with gastric slow-wave rhythms, suggesting a gastric-selective pattern of subcortical–gastrointestinal functional relationship. By integrating central neuroimaging and peripheral electrophysiological measures, these findings delineate distinct neural and visceral substrates underlying autonomic dysfunction in PD. Although the observed relationships are derived from cross-sectional data and therefore cannot establish causality or direct therapeutic implications, they provide a foundation for future longitudinal studies. Such investigations will be essential to determine whether these combined neuro-electrophysiological signatures track the progression of autonomic impairment and its clinical trajectory over time.

## Figures and Tables

**Table 1:** Demographic and clinical characteristics of the participants.

Characteristic	PD (*n* = 23)	HC (*n* = 25)	Test Statistic (t/χ2/Z)	*p*-Value
Gender (Male/Female)	14/9	15/10	χ2 = 0.00	1
Age (years)	71.00 ± 6.65	69.40 ± 6.21	t = 0.94	0.351
BMI (kg/m^2^)	22.74 ± 0.86	22.36 ± 1.57	Z = 353.00	0.18
Education (years)	6.22 ± 3.69	8.44 ± 5.90	Z = 235.50	0.286
MoCA score	24.30 ± 1.55	24.96 ± 1.34	Z = 216.00	0.134
HAMA score	5.22 ± 2.61	4.24 ± 1.45	t = 1.62	0.112
HAMD score	5.09 ± 3.20	4.00 ± 1.87	Z = 342.50	0.255
Mean FD (mm)	0.038 ± 0.01	0.037 ± 0.01	t = −0.825	0.414
Onset Side (Left/Right)	9/14	N/A	N/A	N/A
Disease duration (years)	5.28 ± 4.27;4 (3–7)	N/A	N/A	N/A
Hoehn and Yahr stage	2.74 ± 1.09	N/A	N/A	N/A
MDS-UPDRS-III score	39.17 ± 16.68	N/A	N/A	N/A
LEDD (mg/day)	360.87 ± 191.71	N/A	N/A	N/A
NMSS total score	34.48 ± 12.28	N/A	N/A	N/A
NMSS-GI subscore	3.26 ± 1.57	N/A	N/A	N/A

Note: Continuous variables are presented as mean ± standard deviation (SD); disease duration is additionally reported as median (interquartile range, IQR) due to skewed distribution. Categorical variable (gender) is shown as counts. Normality and homogeneity of variances were examined using the Shapiro–Wilk test and Levene’s test, respectively. For the three continuous variables analyzed by Student’s *t*-test (Age, HAMA, and Mean FD), Levene’s test confirmed homogeneity of variances (all *p* > 0.05). A rank-transformed multivariate analysis of variance (MANOVA) was used to simultaneously compare seven continuous baseline variables and control Type I error inflation (Omnibus Wilks’ lambda = 0.8113, F (7, 40) = 1.3289, *p* = 0.2623). Independent two-sample *t*-tests were used for normally distributed continuous data, Mann–Whitney U tests for non-normally distributed continuous data, and the chi-square test for categorical variables; MoCA, Montreal Cognitive Assessment; HAMA, Hamilton Anxiety Rating Scale; HAMD, Hamilton Depression Rating Scale; UPDRS III, Unified Parkinson’s Disease Rating Scale Part III; LEDD, levodopa equivalent daily dose; NMSS, Non-Motor Symptoms Scale; NMSS-GI, Non-Motor Symptoms Scale gastrointestinal subscore; N/A, not applicable.

**Table 2:** Comparative analysis of EGEG parameters between PD and HC.

Metrics	HC (*n* = 25)	PD (*n* = 23)	F-Value	*p*_unc_-Value	*p*_FDR_-Value	Partial Eta Squared (η_p_^2^)
Preprandial NSW % (1–4)	60.51 ± 1.58	53.16 ± 1.19	16.597	0.0002	**0.0026**	**0.293**
Preprandial NSW % (5–8)	50.24 ± 2.00	40.54 ± 2.55	14.373	0.0005	**0.003**	**0.264**
Preprandial DF (1–4)	2.97 ± 0.04	2.93 ± 0.05	0.166	0.6855	0.6855	0.004
Preprandial DF (5–8)	13.11 ± 0.58	13.63 ± 0.79	1.568	0.2178	0.348	0.038
Preprandial LTD (1–4)	0.50 ± 0.14	0.44 ± 0.16	0.429	0.516	0.563	0.011
Preprandial LTD (5–8)	0.48 ± 0.16	0.18 ± 0.07	2.785	0.1029	0.2471	0.065
Postprandial NSW % (1–4)	64.76 ± 1.81	57.35 ± 1.38	6.494	0.0148	**0.0443**	**0.14**
Postprandial NSW % (5–8)	52.48 ± 1.72	44.47 ± 2.25	10.756	0.0022	**0.0086**	**0.212**
Postprandial DF (1–4)	2.97 ± 0.04	3.06 ± 0.05	1.794	0.188	0.348	0.043
Postprandial DF (5–8)	11.12 ± 0.67	11.23 ± 0.57	0.819	0.3709	0.4451	0.02
Postprandial LTD (1–4)	0.46 ± 0.11	0.39 ± 0.22	1.473	0.232	0.348	0.036
Postprandial LTD (5–8)	0.63 ± 0.17	0.49 ± 0.23	1.262	0.2681	0.3574	0.031

Note: Continuous variables are expressed as mean ± standard error of the mean (SE) to appropriately represent the precision of the inferential statistics. Statistical comparisons between the Parkinson’s disease (PD) and healthy control (HC) groups were performed using analysis of covariance (ANCOVA), strictly adjusting for age, sex, body mass index (BMI), Hamilton Anxiety Rating Scale (HAMA), Hamilton Depression Rating Scale (HAMD), and Montreal Cognitive Assessment (MoCA) scores. Multiple comparisons across the 12 EGEG metrics were corrected using the False Discovery Rate (FDR) method (Benjamini–Hochberg procedure). Statistically significant values, pFDR values (<0.05), are highlighted in bold. Partial Eta Squared (η_p_^2^) was calculated to represent the effect size of the group differences, with values > 0.14 indicating a large effect size. Abbreviations: EGEG, electrogastroenterography; PD, Parkinson’s disease; HC, healthy control; NSW, normal slow wave; LTD, lead-time difference; DF, dominant frequency; FDR, false discovery rate.

**Table 3:** Significant alterations in FC between PD and HCs.

Seeds	Regions	Cluster Size (Voxels)	Peak MNI Coordinates (x, y, z)	Peak t-Value	AAL	BA
L-thalamus	R-insula	42	33, 18, −12	4.82	Insula_R	13
L-caudate	R-caudate	38	12, 6, 9	−5.6	Caudate_R	48
R-putamen	L-putamen	38	−24, 9, −3	−5.15	Putamen_L	49
L-putamen	R-putamen	57	27, 12, −3	−4.94	Putamen_R	49
L-pallidum	L-PCG	55	−6, 9, 51	−5.87	Supp_Motor_Area_L	6

Note: Statistical thresholds were established at a voxel-level significance of *p* < 0.001 and a cluster-level threshold of *p* < 0.05, corrected for false discovery rate (FDR) using two-tailed tests. Abbreviations: FC, functional connectivity; PD, Parkinson’s disease; HCs, healthy controls; MNI, Montreal Neurological Institute; AAL, Automated Anatomical Labeling; BA, Brodmann area; L, left; R, right; PCG, precentral gyrus.

## Data Availability

The datasets used and/or analyzed during the current study are not publicly available due to patient privacy and institutional restrictions, but are available from the corresponding author on reasonable request.

## Supplementary Materials

The following supporting information can be downloaded at: https://www.mdpi.com/article/10.3390/brainsci16080823/s1, Table S1: Variance Inflation Factor (VIF) diagnostic for clinical and demographic covariates; Table S2: Exploratory comparison of functional connectivity (z-values) between left-onset and right-onset PD subgroups; Table S3: Multivariate omnibus effects and assumption diagnostics for the complete MANCOVA model; Table S4: Multivariate omnibus MANCOVA effects for the five functional connectivity metrics; Table S5: Summary of tested hypotheses, statistical models, and multiple comparison corrections across the full analytic pipeline; Text S1: Exploratory Subgroup Analysis of Motor Symptom Lateralization; Text S2: Rank-Transformed Multivariate Analysis of Covariance (MANCOVA); Text S3: Detailed Justification of Sample Size, Statistical Power, and Effect Sizes; Text S4: Detailed Technical Parameters of rs-fMRI Preprocessing and Multi-Modal Acquisition.

## Abbreviations

AAL	Automated Anatomical Labeling
ART	Artifact Detection Tool
BA	Brodmann area
CAU	Caudate
PD	Parkinson’s Disease
DF	Dominant frequency
EGEG	Electrogastroenterography
FC	Functional connectivity
HAMA	Hamilton Anxiety Rating Scale
HAMD	Hamilton Depression Rating Scale
HC	Healthy control
GID	Gastrointestinal dysmotility
INS	Insula
LEDD	Levodopa equivalent daily dose
MoCA	Montreal Cognitive Assessment
NSW	Normal slow wave
PAL	Pallidum
PCG	Precentral gyrus
PUT	Putamen
THA	Thalamus
UPDRS-III	Unified Parkinson’s Disease Rating Scale Part III
