# Supplementary material for: Gastric-Selective Associations of Caudate Functional Connectivity with Gastrointestinal Rhythms in Parkinson’s Disease: A Resting-State fMRI and Electrogastroenterography Study
Source: Brain Sci. 2026 Aug 1;16(8):823. doi: 10.3390/brainsci16080823 (PMC13510822; doi:10.3390/brainsci16080823)
Supplement: Supplementary file 1 [file brainsci-16-00823-s001.zip › brainsci-4355387-supplementary.pdf]

Gastric-selective associations of caudate functional connectivity with gastrointestinal rhythms in Parkinson's disease: A resting-state fMRI and electrogastroenterography study

Zhining Li[1,2], Nana Shen[2], Can Li[2], Liangqun Rong[2], Zhengwei Chen[1,2], Chun-feng Liu[1,]

Table S1. Variance Inflation Factor (VIF) diagnostic for clinical and demographic covariates.

Covariates	VIF
Age	2.41
Gender	1.31
BMI	1.78
LEDD	1.85
HAMA	1.37
HAMD	1.78
MoCA	1.92
Mean FD	1.88

Note: All Variance Inflation Factor (VIF) values are well below the conservative threshold of 5, indicating no significant multicollinearity among the selected covariates in the partial correlation analysis.

Supplementary Text S1. Exploratory Subgroup Analysis of Motor Symptom Lateralization

This study was based on the fact that dopaminergic neuron depletion in Parkinson's disease exhibits characteristic asymmetry. We conducted an exploratory subgroup analysis to preliminarily assess patients with left-sided onset (n=9) and right-sided onset (n=14). The results revealed potential differences in central functional reorganisation among patients with Parkinson's disease.

Preliminary results indicate that there were significant differences across subgroups in the functional connectivity between the left globus pallidus and the left precentral gyrus ($t = 3.290$, $p = 0.003$), as well as between the left thalamus and the right insula ($t = -3.001$, $p = 0.007$) (Table S2). Importantly, there were no significant differences detected in interhemispheric striatal connections, including those involving the bilateral caudate nuclei, nor in the connection between the caudate nuclei (left CAU-right CAU) ($t = -1.997$, $p = 0.058$). However, in our main analysis, this connection was identified as being specifically coupled to the gastric pacemaker rhythm.

Limitation of Exploratory Analysis

Limitations of exploratory analysis: This study emphasises that the subgroup analyses are exploratory in nature. Their aim is to generate hypotheses. Given the extremely small sample sizes in each subgroup (n=9 and n=14), the statistical power is insufficient to demonstrate lateralisation of functional connectivity. These preliminary findings are highly susceptible to extreme inter-individual variability and carry a high risk of Type I errors. The lateralisation trends observed here should be interpreted with extreme caution. In the future, we will conduct independent studies using larger, demographically balanced cohorts to validate these effects.

Table S2. Exploratory comparison of functional connectivity (z-values) between left-onset and right-onset PD subgroups.

FC Regions (Seeds to Results)	Left-onset PD (n = 9)	Right-onset PD (n = 14)	t-value	p-value	Levene'sF	p.Levene
Left PAL-Left PCG	0.008 ± 0.115	−0.070 ± 0.085	3.290	**0.003**	0.5945	0.4493
Left THA-Right INS	0.030 ± 0.132	0.166 ± 0.081	−3.001	**0.007**	**0.5738**	**0.4572**
Left CAU-Right CAU	0.194 ± 0.073	0.259 ± 0.085	−1.997	0.058	10.4496	0.0040
Left PUT-Right PUT	0.101 ± 0.150	0.189 ± 0.139	−1.511	0.145	0.1080	0.7457
Right PUT-Left PUT	0.145 ± 0.153	0.225 ± 0.153	−1.133	0.270	0.6007	0.4469

Note: Data are expressed as mean ± standard deviation (SD). Inter-subgroup differences in the extracted Fisher-transformed z-values were evaluated using independent two-sample t-tests. Values with $p < 0.01$ are highlighted in bold. Caution: Given the small sample size (n=9 vs n=14),this analysis is statistically underpowered and the results should be considered exploratory. Abbreviations: FC, functional connectivity; PD, Parkinson's disease; PAL, pallidum; PCG, precentral gyrus; THA, thalamus; INS, insula; CAU, caudate; PUT, putamen.

Supplementary Text S2. Rank-Transformed Multivariate Analysis of Covariance (MANCOVA)

Following the rank transformation technique (Conover & Iman, 1981), the omnibus multivariate test (adjusting for age, sex, BMI, MoCA, HAMA, and HAMD) confirmed a highly significant global difference in the spatiotemporal EGEG profile between the Parkinson's disease and healthy

control groups (Wilks' Lambda=0.4762, F(7, 40) =2.6584, P=0.0154). This system-level validation mathematically guarantees that the observed gastrointestinal dysrhythmias are robust and independent of single-test statistical inflation.

Table S3. Multivariate omnibus effects and assumption diagnostics for the complete MANCOVA model.

Source of Variation / Test	Wilks' Lambda / Metric Value	F-value	p-value	Conclusion / Assumption Status
Main Factor: Group (PD vs. HC)	0.4762	2.6584	0.0154	Highly Significant Group Effect
Covariate: Age	0.6759	1.1588	0.3556	Covariate Main Effect
Covariate: Sex	0.8274	0.5041	0.8949	Covariate Main Effect
Covariate: BMI	0.7497	0.8067	0.6415	Covariate Main Effect
Covariate: MoCA	0.5182	2.2471	0.0369	Covariate Main Effect
Covariate: HAMA	0.7666	0.7357	0.7063	Covariate Main Effect
Covariate: HAMD	0.5901	1.6789	0.1242	Covariate Main Effect
Homogeneity of Slopes (Group×Covariates)	0.1177	0.2208	1.0000	Assumption Met (Parallel Slopes)
Box's M Test	93.9991	--	0.1047	Assumption Met (Equal Covariances)

Note: All 12 spatiotemporal EGEG parameter sets were evaluated simultaneously withinthis full-factorial multivariate framework. The non-significant interaction term (Group × Covariates, $p > 0.05$) statistically validates the mathematical appropriateness of ourcovariate-adjusted baseline profiles.

Table S4. Multivariate omnibus MANCOVA effects for the five functional connectivity metrics (Figure 5).

Source of Variation (Omnibus Test)	Wilks' Lambda	F-value	p-value	Statistical Conclusion
Main Factor: Group (PD vs. HC)	0.1890	28.3255	<0.001	Highly Significant Systemic FC Difference
Age (Covariate)	0.9102	0.6514	0.6625	Covariate Effect
Sex (Covariate)	0.7513	2.1849	0.0796	Covariate Effect
BMI (Covariate)	0.9320	0.4817	0.7873	Covariate Effect
MoCA (Covariate)	0.9034	0.7061	0.6230	Covariate Effect
HAMA (Covariate)	0.7802	1.8594	0.1285	Covariate Effect
HAMD (Covariate)	0.9360	0.4516	0.8090	Covariate Effect
Mean FD (Covariate)	0.9322	0.4803	0.7883	Covariate Effect

Supplementary Text S3. Detailed Justification of Sample Size, Statistical Power, and Effect Sizes

Due to the small sample size of the Parkinson's disease cohort in this study (N=23 compared with N=25 healthy controls)， we conducted a rigorous post-hoc statistical power analysis to assess the statistical validity of the primary baseline measures of brain–gut coupling. This study identified a positive correlation between interhemispheric functional connectivity in the caudate nucleus and the percentage of normal slow waves in the stomach following a meal (r =0.630, 95% CI = [0.015, 0.930]). We used G*Power software (version 3.1.9.7) to calculate the statistical power (1–B) achieved. This empirical model achieved 91.2% statistical power under conditions of a two-tailed α =0.05 and a sample size of N=23, which exceeds the international benchmark of 80%. This ensures that the present study possesses sufficient statistical power to reject the null hypothesis and detect a genuine cross-system physiological interaction.

Furthermore, regarding the between-group EGEG parameters evaluated via ANCOVA (Table 2), the calculated effect sizes (Partial Eta Squared, $\eta 2$) for all statistically significant variations ranged from 0.140 to 0.293. According to standard econometric criteria, $\eta^2 p > 0.14$ indicates a large effect size. This implies that the group classification explains between 14.0% and 29.3% of the total variance in peripheral electrophysiological rhythmicity. Overall, the strict 1,000-resampling Bootstrap confidence intervals, the significant effect sizes, and a post-hoc power exceeding 91% mathematically demonstrate that our findings are robust with regard to Type I error inflation. Furthermore, despite the relatively small sample size, the study exhibits sufficient resilience against Type II errors.

Table S5. Summary of tested hypotheses, statistical models, and multiple comparison corrections across the full analytic pipeline.

Pipeline Stage & Target Table/Figure	Core Scientific Hypothesis Checked	Number of Related Outcomes	Primary Statistical Model	Multiplicity Control Strategy (Type I Error Correction)	Final Corrected Significance Threshold
Stage 1: Clinical Characteristics(Table 1)	Demographic baseline match and absence of confounding clinical heterogeneity between PD and HC groups.	7 variables (Age, Sex, BMI, MoCA, HAMA, HAMD, Mean FD)	Independent t-tests / Chi-square ($\chi2$) / Mann-Whitney U	Individual premise screening via Levene's and Shapiro-Wilk tests; no global correction enforced as analysis is purely descriptive of cohort heterogenity.	Uncorrected α=0.05 (justified as descriptive baseline profiling)
Stage 2: Electrophysiology(Table 2)	Systemic degradation of spatiotemporal gastric and colonic slow-wave rhythmicity in patients with PD.	12 separate EGEG parameters	Rank-transformed MANCOVA followed by univariate ANCOVAs	Step 1: Top-down multivariate omnibus validation.Step 2: False Discovery Rate (FDR) correction across the 12 univariate models.	MANCOVA Omnibus: p<0.05Univariate ANCOVA: pFDR <0.05
Stage 3: Neuroimaging(Figure 5 & Table 3)	Functional disruption of subcortical networks regulating distinct cortico-basal ganglia-thalamocortical circuits in PD.	Whole-brain search matrices across 8 predefined subcortical seeds	Seed-to-voxel whole-brain functional connectivity (FC)	Layer 1 (Spatial): Whole-brain voxel-level screening combined with Cluster-level FDR correction.Layer 2 (Cross-Seed): Post-hoc strict Bonferroni penalty across all 8 independent seed channels.	Voxel-level: p<0.001 (uncorrected)Cluster-level: pFDR <0.05Cross-Seed Barrier: pCluster-FDR <0.05/8=0.00625
Stage 4: Brain-Gut Mapping(Table 4)	Synchronous multi-system functional	20 unique brain-gut contrast	Partial correlation analysis with	Localized, hypothesis-driven per-seed	Localized per-seed barrier:puncorrected <0.05/4=0.0125

Pipeline Stage & Target Table/Figure	Core Scientific Hypothesis Checked	Number of Related Outcomes	Primary Statistical Model	Multiplicity Control Strategy (Type I Error Correction)	Final Corrected Significance Threshold
	coupling between altered subcortical networks and peripheral EGEG rhythmicity.	vectors	bootstrap validation	Bonferroni correction (4 contrasts per seed matrix) to balance Type I and Type II error inflation.	
Stage 5: Clinical Correlation(Table 5 & Figure 6)	Clinical-behavioral relevance of central network dissolution on non-motor symptoms and general disease severity.	15 unique brain-behavior contrast vectors	Partial correlation analysis with regression diagnostics	Localized, circuit-specific Bonferroni correction (3 clinical targets per functional network cluster).	Localized per-circuit barrier:puncorrected <0.05/3=0.0167

Note: EGEG, electrogastroenterography; FC, functional connectivity; PD, Parkinson's disease; HC, healthy controls; FDR, False Discovery Rate; MANCOVA, Multivariate Analysis of Covariance; ANCOVA, Analysis of Covariance.

Supplementary Text S4. Detailed Technical Parameters of rs-fMRI Preprocessing and Multi-Modal Acquisition

Section 2.4 rs-fMRI Preprocessing and Spatial Smoothing Pipeline

Functional imaging data were pre-processed using SPM12 and the CONN toolbox in MATLAB (version 18b). To ensure maximum methodological rigour and transparency, our analysis workflow strictly adhered to the standardised resting-state fMRI clinical mapping consensus guidelines endorsed by the American Society of Functional Neuroimaging [1]. We followed the pre-processing steps explicitly recommended for seed-based correlation (SBC) analysis. Following slice-time alignment and progressive rigid-head motion realignment, the time-series data underwent specialised wavelet de-spiking to truncate transient non-physiological intensity

changes. Subsequently, functional voxels have been normalised to the Montreal Neurological Institute (MNI) space, with an unsmoothed isotropic voxel size of $3 \times 3 \times 3$ mm³. Time filtering was performed using a defined band-pass filter (0.01–0.08 Hz) to eliminate low-frequency scanner drift and high-frequency physiological noise. Importantly, our study strictly adhered to consensus requirements stipulating that the spatial smoothing kernel size should be twice the size of the effective voxel to optimise signal-to-noise ratio separation whilst maintaining the topological field assumption.

Section 2.5 Nuisance Regression, Seed Definition, and Thresholding

In order to effectively isolate interference in blood oxygen level-dependent (BOLD) network signals caused by pseudo-motion and systemic non-neural variations, this study implemented a standardised multi-level interference regression framework in accordance with the SBC guidelines [1]. We performed dynamic volume truncation (erasure) using the Artifact Detection Tool (ART), whereby frames with frame-to-frame displacement (FD) exceeding 0.9 mm or global BOLD changes exceeding 5 standard deviations were identified and masked.

To eliminate the influence of non-neural tissue, an anatomy-component-based noise correction (aCompCor) method was employed. This study performed interference regression on the time curves of cerebrospinal fluid (CSF) and white matter (WM) signals extracted from segmented structural masks. Furthermore, we rigorously applied a standard 24-parameter Friston autoregressive model, derived from image registration, for head motion regression to eliminate residual distance-dependent connectivity biases.

The eight subcortical seeds within the striatal-thalamic network were automatically segmented using the Harvard-Oxford probabilistic subcortical atlas natively integrated within the CONN

toolbox. Group-level seed-to-voxel correlations were mapped using a layered topologicalthresholding strategy: an initial voxel-level screening of uncorrected $p<0.001$, combined with a cluster-level False Discovery Rate (FDR) correction threshold of $pFDR < 0.05$ across the entire brain space.

References

1.Kumar, V.A.; Lee, J.; Liu, H.L.; Allen, J.W.; Filippi, C.G.; Holodny, A.I.; Hsu, K.; Jain, R.; McAndrews, M.P.; Peck, K.K.; et al. Recommended Resting-State fMRI Acquisition and Preprocessing Steps for Preoperative Mapping of Language and Motor and Visual Areas in Adult and Pediatric Patients with Brain Tumors and Epilepsy. AJNR. *Am. J. Neuroradiol.* **2024**, *45*, 139–148. https://doi.org/10.3174/ajnr.A8067.
